# Characteristics and Effects of Outdoor Activities on People Living with a Major Neurocognitive Disorder: A Scoping Review

**DOI:** 10.1177/19375867251412441

**Published:** 2026-02-17

**Authors:** A. Laberge, M. Veillette, A. Ayotte, M. Taillefer, C. Viscogliosi

**Affiliations:** 1 7321 Université de Sherbrooke, Sherbrooke, Canada; 2 7321 Université de Sherbrooke et Centre de recherche sur le vieillissement du CIUSSS de l’Estrie-CHUS, Sherbrooke, Canada

**Keywords:** outdoor activity, older adult, dementia, qualitative research, scoping review

## Abstract

**Objectives:**

Considering the lack of cure for MNCD, this article focuses on non-pharmacological interventions such as outdoor activities, their characteristics and their effects on behaviors, symptoms, quality of life, autonomy and cognitive functioning.

**Background:**

People living with major neurocognitive disorders (MNCD) may experience reactive behaviors, symptoms, decreased autonomy, quality of life and cognitive functioning. There is currently no treatment that can reverse or halt the cognitive decline resulting from MNCD.

**Methods:**

A scoping review was conducted using the method of Arksey and O'Malley (2005) and following PRISMA guidelines. AgeLine, APA PsycInfo, CINAHL Plus with Full Text and MEDLINE were used to conduct the literature search.

**Results:**

Most outdoor activities identified in this study were: activities carried out in a garden (n=5); activities involving active participation (n=14); activities offering the possibility of being carried out in a group or individually (n=6); activities lasting less than an hour (n=7); and activities taking place in the participants' place of residence (n=13). Most of studies showed that outdoor activities were associated with positive impacts on overall mood (n=12). Several studies reported reduced agitation (n=7), improved cognitive functioning (n=5) and increased well-being (n=5) in participants undertaking various outdoor activities.

**Conclusions:**

Outdoor activities and the associated freedom to make decisions led to a range of benefits in the five categories that were studied. These benefits apply whether the activities are carried out in a group or individually, whether they are active or inactive, and whether they take place close to home. Future research would be relevant to specify the characteristics of the activities to be performed according to the desired benefits.

## Introduction

The ageing of the Canadian population is accompanied by an increase in the prevalence of major neurocognitive disorders (MNCD) also called dementia (Alzheimer Society of Canada, 2024a; Alzheimer Society of Canada, 2024c). In fact, in 2020, 8.4% of Canadians over 65 were living with an MNCD. It is estimated that by 2050, this proportion could rise to 13.2%. By 2030, nearly 1 million Canadians will be living with dementia, and more than 1.7 million by 2050. Alzheimer's disease is the most prevalent MNCD that also includes vascular neurocognitive disorders, frontotemporal degeneration, Lewy body, and other forms of MNCDs (Sheppard & Coleman, 2020). Decreased cognitive functioning and autonomy resulting in difficulties remaining at home are among the major impacts that can result from MNCD. In fact, memory, thinking, language, and problem-solving difficulties, most often resulting from MNCD, affect a person's ability to carry on with daily activities (Alzheimer Society of Canada, 2024c). Furthermore, MNCD can lead to different consequences such as a change in behaviors (e.g., agitation, wandering) and appearances of symptoms (e.g., mood swings, varying levels of wakefulness). Moreover, people living with MNCD are often unable to meet their needs independently or communicate them clearly to others, which can adversely affect their quality of life (Bourbonnais et al., 2021). Thus, it appears essential to find ways to intervene more effectively with this clientele in order to meet the needs of the growing number of people living with an MNCD.

Despite the lack of a cure for MNCDs (Alzheimer Society of Canada, 2024b), there are alternative means of slowing the progression of the disease and reducing its impact. Among these, environmental design (e.g., camouflaging doors or doorknobs) shows positive impacts on behavioral and psychological symptoms of dementia (BPSD) (Bonin et al., 2020). Cognitive stimulation is also among the non-pharmacological treatments with benefits for cognitive functioning and quality of life in people with MNCD (Alvares Pereira et al., 2022).

Across a range of health conditions, outdoor activity shows positive effects on affect, cognition, well-being, and symptoms of anxiety and depression (Lackey et al., 2021). In addition, a scoping review of the effects of nature-based rehabilitation found that it improved motor and sensory-motor function, quality of life, and cognitive function in people with brain injury (stroke, CBT, and neurotoxic accident) (Vibholm et al., 2020). To our knowledge, no study has documented the benefits of outdoor activities and their impact-related characteristics specifically for people living with MNCD. In order to contribute to the improvement of behaviors, symptoms, quality of life, and autonomy of people living with MNCD, it appears important to explore the possible impacts of outdoor activities specifically for people living with MNCD as well as the characteristics of these activities. Thus, this scoping review will identify and describe the characteristics of outdoor activities and their effects on the behaviors, symptoms, quality of life, autonomy, and cognitive functioning of people living with MNCD.

## Methodology

**Figure 1.**
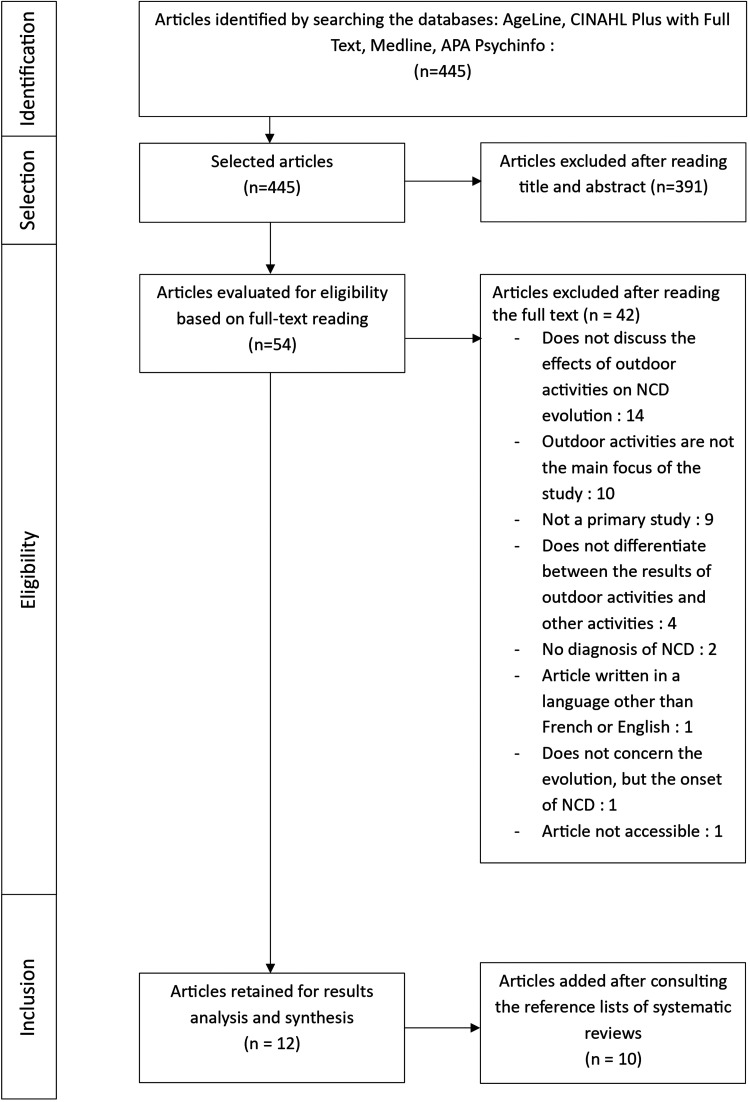
Flowchart.

This scoping review followed the methodology proposed by Arksey and O’Malley (2005), which consists of five stages: identification of the research question, identification of relevant studies, selection of studies, extraction and classification of data, and finally, synthesis of data and communication of results. PRISMA guidelines were also followed (Figure 1). The aim of this methodology is to examine the extent and nature of research related to the question posed, to synthesize and disseminate research findings, and to identify gaps in the literature.

### Identifying the Research Question

The research question selected for this study is as follows: What are the characteristics of outdoor activities and their effects on the behaviors, symptoms, quality of life, autonomy, and cognitive functioning of people living with MNCD?

### Identifying Relevant Studies

The literature search was carried out on January 31, 2024, and updated on October 24, 2024. The search strategy was based on a concept plan whose keywords were determined by the entire research team (Table 1). This strategy was validated and improved by an expert librarian in the healthcare field. Four databases were consulted: AgeLine, APA PsycInfo, CINAHL Plus with Full Text and MEDLINE. Selected keywords were searched in document titles and abstracts.

**Table 1.** Literature Search Strategy.

Concepts	Keywords
Neurocognitive disorders	neurocognitive disorder*” OR NCD OR dementia* OR Alzheimer* OR “cognitive disorder*” OR “cognitive impairment*” OR “organic mental disorder*” OR “organic brain syndrome*.”
Outdoor activities	(outdoor* OR exterior* OR natur* OR outside) N3 (activit* OR recreation* OR time OR exercic* OR hobb* OR leisure*)

### Selection of Studies

Search results were exported to Zotero, a bibliographic reference management software, where duplicates were removed. To be selected, publications had to meet the following inclusion criteria: (1) The study population had an MNCD. Studies in which caregivers or family members reported effects on people living with MNCD were included. (2) Selected studies had to include one or more outdoor activities in which individuals participated actively (e.g., going for a walk) or inactively (e.g., sitting in a garden). These activities had to take place outside of a building. Articles dealing with activities taking place outside the home but taking place in an indoor environment (e.g., grocery shopping, visiting a fitness center) were not considered. In addition, articles mentioning activities that are typically performed outdoors but were carried out indoors (e.g., gardening indoors) were not retained for this study. (3) Only empirical studies were included (primary studies). (4) The publication had to report qualitative and/or quantitative results related to the research question. (5) The publication date was between 2009 and 2024. In addition, publications were excluded if they met the following exclusion criteria: (1) Studies in which participants had cognitive deficits without a diagnosis of MNCD. (2) There were no results linking outdoor activities to MNCD or its manifestations. (3) Outcomes associated with outdoor activities were not dissociable from those associated with indoor activities. (4) The article was written in a language other than English or French.

To select the articles relevant to the research, we first formed two dyads within the research team. The articles were divided equally and randomly between the dyads, with each reviewer individually analyzing the title and abstract according to inclusion and exclusion criteria. The dyads then pooled their findings to identify any differences in the articles selected. A discussion between the members of the dyad resolved any differences. If a disagreement persisted after the discussion, all members of the research team were consulted at a meeting to validate the selection of articles, and a joint decision was taken. After this consultation, if ambiguity persisted, the articles were included for full-text reading. The second step in the article selection process was to read the full texts to assess whether they should be retained. New dyads were formed among the members of the research team, and the articles selected for full reading were distributed equally and randomly among them. The same method as in the first stage, starting with an individual analysis, was applied to the inclusion of articles and to the resolution of ambiguities. Ambiguities were resolved at a validation meeting involving all members of the research team. Subsequently, articles included in the reference list of four systematic reviews and a scoping review meeting the inclusion criteria were considered. At the end of this process, 22 articles were included in the scoping review.

### Data Extraction and Classification

The extraction of data relevant to answering the research question was carried out following a complete reading of the articles. This identified the authors, date of publication, country, type of study, specifications, objectives, and data collection method (Table 2). The extraction also highlighted the characteristics of the outdoor activities (type of activity, context, frequency, duration, and location of activity), the characteristics of the study population, the effects observed on the autonomy, symptoms, behaviors, quality of life, and cognitive functioning of people living with MNCD, and finally, the limitations and recommendations of the studies (Table 3). These characteristics were chosen because they were the main variables investigated by the selected studies. The results reported in the reviewed studies concern both decision-making autonomy and functional autonomy. With regard to symptoms, the results of the studies reviewed included: mood, affect, level of arousal, feelings of loneliness, depression, happiness, levels of distress, energy levels, emotional changes, disinhibition, apathy, dysphoria, euphoria, hallucinations, delusions, and so on. On the other hand, behavioral findings in the studies reviewed included: agitation, social interactions and isolation, aggressive behaviors, communication (patient-patient, and patient-caregiver), resistance to care, wandering, repetition (words/questions/activities), expressions of suspicion, etc. For quality of life, results included perception of well-being and satisfaction with social relationships. Finally, results dealing with memory, visuospatial skills, executive functions, attention, concentration, working memory, language skills, temporal and spatial orientation, learning, reminiscence, problem solving, anosognosia, judgment, introspection, and body representation were extracted in relation to cognitive functioning. Data extraction was carried out individually. Two data extraction validation meetings were held at the beginning and end of the process, to ensure standardization.

**Table 2.** Description of Selected Studies.

Authors (year)	Country	Type of study	Study design	Study objectives	Data collection method
Beerens et al. (2018)	Netherlands	Quantitative	Observational	To assess the association between mood, activity engagement, activity location and social interaction during daily life of people living with NCD residing in long-term care facilities.	- Maastricht Electronic Daily Life Observation Tool (MEDLO).
Borella et al. (2023)	Italy	Quantitative	Pilot test	To evaluate the effects of horticultural activities on people living with NCD in a residential and day care setting by examining benefits on general cognitive functioning, mood, behavioural and psychological symptoms, and quality of life. Understand the effects and issues associated with horticultural activities. Examine whether and to what extent the inclusion of features and elements drawn from other effective psychosocial interventions for NCD (e.g., cognitive stimulation therapy) could promote the benefits offered by horticultural activities.	Tests and questionnaires: - Montreal Cognitive Assessment (MoCA). - Alzheimer's Disease Assessment Scale-Cognitive subscale (ADAS-Cog). - Cornell Scale for Depression in Dementia. - Neuropsychiatric Inventory (NPI). - Quality of Life-Alzheimer's Disease scale (QoL-AD).
Bourdon and Belmin (2021)	France	Quantitative	Pilot test	To evaluate the benefits of an enriched environment, applied to gardens in residences for the elderly, on clinical markers of function in residents living with NCD.	- Mini-Mental State Examination (MMSE) - Activities of daily living (ADL) - Timed up and go (TUG) - Unipodal positioning test
Buettner et al. (2013)	United States	Mixed	Randomized clinical trial (secondary analysis)	To assess the effectiveness of cycling activity compared with usual interventions on effects on engagement, mood and need for encouragement to participate in activity. The experimental intervention involved placing patients in a wheelchair attached to the front of a bicycle, then having them ride an outdoor customized circuit by a staff member.	- 7-point Global Deterioration Scale, - Farrington Leisure Interest Survey. - Interviews with the participant, his/her family and staff providing direct care to the participant.
Cedervall and Aberg (2010)	Switzerland	Qualitative	Observational descriptive	To explore the significance of the lived experiences of people living with Alzheimer's type NCD and their loved ones, in terms of the physical activities that are practised by people living with NCD.	- Qualitative interviews - Comments from participants
Cook (2020)	United Kingdom	Qualitative	Pilot test	To understand how the use of woodlands and urban forests can contribute positively to the mental well-being of people living with NCD, in particular by exploring the meaning attached to the practice of this activity by the target population.	- Semi-structured interviews. - Observations.
Edwards et al. (2013)	Australia	Mixed	Convergent	To evaluate the effects of setting up a therapeutic garden on the quality of life of elderly people living with NCD.	- DEMQOL/DEMQOLProxy (Dementia Quality of Life Instrument), - CSDD (Cornell Scale for Depression in Dementia) - CMAI (Cohen-Mansfield Agitation Inventory) - Mini-Mental State Examination (MMSE). - Interviews with staff and family members.
Evans et al. (2022)	United Kingdom	Mixed	Pilot test	Give people living with NCD opportunities and support to access nature-based environments to improve health and well-being. Measure the effects of the project by exploring the implementation process, the outdoor activities proposed and the effects on people living with NCD and their caregivers.	- Interviews with participants, caregivers and practitioners. - Modified version of the Shorter Warwick-Edinburgh Mental Well-being Scale (SWEMWBS) to help people living with NCD understand their response choices. - Self-reported questionnaire on the number of physical activity sessions per week.
Gueib et al. (2020)	France	Quantitative	Observational	To evaluate the impact of having access to a healing garden on a clientele living with NCD during hospitalization. To assess the impact of hospitalization on the self-awareness of patients with major neurocognitive disorders hospitalized in Cognitive Behavioural Units, and to learn about the impact of a specific environment made available to these patients, namely the *Art, Memory and Life* garden*.*	− Self-consciousness questionnaire (SCQ). - Mini-Mental State Examination (MMSE). - Mini Geriatric Depression Scale (mini-GDS) - Rapid Frontal Assessment Battery (FAB) - Neuropsychiatric Inventory - Nursing Homes (NPI-NH) - Montreal-Toulouse Protocol 86 module 1α (MT86-1α) - The care team takes note of the time patients spend in the garden. - Observations by the care team.
Ibsen et al. (2020)	Norway	Mixed	Observational	To describe the characteristics of people living with NCD attending farm-based day care (FDC) in Norway. To explore whether the individual characteristics of participants and the agricultural characteristics of the CDF are associated with quality of life.	- Standardized assessment forms and information provided by the person him/herself or his/her caregiver to assess the health characteristics of people living with NCD. - Quality of Life-Alzheimer's Disease Scale. - Oslo Social form. - Montreal Cognitive Assessment (MoCA). - Clinical Dementia Rating Scale - Anosognosia assessment scale. - Depression rating scale. - Norwegian version of Rating Anxiety in Dementia. - Neuropsychiatric Inventory. - General Medical Health scale. - Euro-QoL visual analogue scale - Physical Self-Maintaining Scale. - ADL Instrumental Scale. - Interviews with participants and their next of kin.
Levinger et al. (2022)	Australia	Quantitative	Feasibility study	Adapting the *Senior Exercise Park* program to people living with mild to moderate NCD, to enable them to engage in physical and social activities.	Measuring mood and pleasure through: - 5-point Likert scale - Visual maps with faces - UCLA 3-Item Loneliness Scale - Lubben Social Network Scale (LSNS-6) - Quality of Life in Alzheimer's disease scale (QoL-AD) - EQ-5D-5L with visual analogue scale
Liao et al. (2020)	United States	Quantitative	Pilot test	Evaluate the effects of garden visits on mood, social interaction, cognition and behavioural problems. Identify the types of behavioural problems and cognitive skills that can be improved in patients living with NCD after visiting a garden.	- Semi-structured questionnaire completed by staff members. - Observations of cognitive and behavioural characteristics and changes in participants.
MacAndrew et al. (2019)	Australia	Qualitative	Feasibility study	Explore the demand for a program for residents living with severe NCD and wandering in a CHSLD home. Verify the feasibility of implementing the *Safe Walking Program* (SWP) for this clientele. Understand staff perceptions of SWP use in a CHSLD home and barriers to its sustained use.	- Staff interviews - Checklist completed by a trained operator and a second observer after each walk.
Mapes (2012)	United Kingdom	Mixed	Pilot test	Gather qualitative and quantitative evidence about the benefits of woodland activities for people living with NCD in care home settings. Develop digital material to inspire and inform other excluded groups to consider doing activities in the woods.	- Participatory evaluation questionnaire. - Data collection via graph and box drawings on large flip charts, with participants placing stickers and notes with comments in the relevant boxes.
Ford Murphy et al. (2010)	United States	Quantitative	Observational	To evaluate the effect of visiting the garden on the agitation scores of elderly people living with NCD as a function of the locomotor aids used.	- Short form of the Cohen-Mansfield Agitation Inventory (CMAI) - The number of visits to the garden. - Each patient's ambulatory condition.
Olsson et al. (2013)	Sweden	Qualitative	Phenomenological	Describe the perceptions of people living with NCD regarding the benefits of time spent outdoors. Understand the experiences and reflections of study participants.	- Interviews with participants at two points in time, including at least one interview without the presence of family members. - Home and neighborhood observation. - Observation of participants’ non-verbal behaviour.
Prolo and Sassi (2017)	Switzerland	Mixed	Observational quasi-experimental	Evaluate the impact of odours diffused in a garden on the frequency of reactive behaviours such as wandering, agitation, anger and anxiety. Evaluate the effect of age and gender on the frequency of individual behaviours. Describe participants’ reactions to particular aspects: ramps, sculptures, fountains, frequency of falls and urges to urinate.	- Semi-structured interviews with participants. - Caregiver Burden Assessment questionnaire.
Ross et al. (2024)	Germany	Qualitative	Phenomenological	Document how family caregivers manage the implementation of non-pharmacological approaches, what the associated effects are, how these approaches are funded and what the barriers to their integration are.	- Structured interviews with participants.
Smith-Carrier et al. (2021)	Canada	Qualitative	Phenomenological	Explore therapeutic gardening experiences for people living with NCD and their perspectives on the meaning and emotions elicited during gardening. Understand the meaning attributed to gardening by participants and what makes it a valuable experience.	- Semi-structured interviews with participants - Observations during gardening activities at the day centre.
Ura et al. (2021)	Japan	Mixed	Convergent	Establish care farms promoting rice cultivation. To explore the positive effects associated with rice care farms and compare the effects of these farms with usual care on well-being and cognitive function.	- Semi-structured interviews with people living with NCD and staff. - Mini-Mental State Examination (MMSE). - Japanese version of the World Health Organization-Five Well-Being Index (WHO-5-J). - Two question methods to assess the presence of depressive symptoms.
van der Velde-van Buuringen et al. (2021)	Netherlands	Mixed	Feasibility study	To evaluate the process of daily outings to the CHSLD home's outdoor gardens and explore the effects of gardening on quality of life and neuropsychiatric symptoms in people with NCD.	- Interviews with caregivers and professionals. - Questionnaires completed by professionals taking part in the intervention. - Short version of QUALIDEM completed by the caregiver and/or professionals. - Neuropsychiatric Inventory-Nursing Homes (NPI-NH). - Participants’ diaries.
White et al. (2018)	United Kingdom	Quantitative	Observational	To assess the association between duration of exposure to nature and mood changes in people at any stage of NCD.	- Data sheets filled out by caregivers and activity coordinators, reporting residents’ outdoor experiences, month and day of the week, mood before and after the outing, and time spent outdoors.

**Table 3.** Description of Outdoor Activities and Their Effects.

Authors (date)	Type of outdoor activities	Background to outdoor activities	Frequency and duration of outdoor activities	Location of outdoor activities	Participant characteristics	Effects observed on participants	Authors’ limitations and recommendations
Beerens et al. (2018)	Outdoor walking, farming and animal-related activities.	Not specified.	Not specified.	Activities took place inside or outside long-term care facilities. -8% of activities took place outdoors.	n = 115 - 115 people with a diagnosis of NCD. - M = 83.8 years (SD = 7.8). - 75% were women. - 66% were widowers.	**Symptoms**: Highest mood scores = when participants were engaged in nature-related outdoor activities (M = 5.2). - When activities were performed outdoors (M = 5.1), participants had predominantly positive mood scores at 95%. - More positive mood was associated with participating in activities, being outside during activities and having social interactions (p < 0.001).	Limits: - No causal relationship established (associations only). 14 of 18 services = small-scale facilities. Participants living in small-scale facilities may be more engaged in outdoor activities and have more social interaction. Recommendations: - Qualitative studies, preferably randomized intervention studies, are needed to assess how being outdoors and access to outdoor space influence mood, and which specific outdoor activities have the greatest potential for positive mood.
Borella et al. (2023)	- Horticultural activities (planting beds, planting seeds, horticultural species, transplanting, aromatic plants, colourful flowers, shelling beans and other vegetables, harvesting aromatic plants). - Activities graduated from simple (touch, feel, observe) to complex (plant, water).	- Small groups of 3 to 5 people.	- 12 activity sessions - Approx. 40 min, 2 times a week for 6 weeks.	- Therapeutic garden outside the facility. - Easily accessible and visible from inside the facility. - Landscaping with chairs, paving stones and raised planters.	n = 24 TG1: horticultural activity group TG2: horticultural activity group + cognitive stimulation CG: control group participating in usual indoor educational activities. Full sample: TG1: n = 7; mean age = 79.14 (SD = 5.64); years of education = 7.57 (SD = 5.09) TG2: n = 8; mean age = 81.25 (SD = 5.17); years of education = 4.38 (SD = 2.32) CG: n = 9; mean age = 82.67 (SD = 6.91); years of education = 7.88 (SD = 3.64) Participants with mild-to-moderate NCD only: TG1: n = 4; mean age = 79.00 (SD = 4.96); years of education = 9.25 (SD = 6.23) TG2: n = 7; mean age = 81.29 (SD = 5.58); years of education = 4.29 (SD = 2.49) CG: n = 5; mean age = 86.00 (SD = 3.00); years of education = 8.40 (SD = 2.88) –NCD - Autonomous for locomotion or to move around with a mobility aid.	**Behaviours and symptoms:** - TG group vs. CG group = fewer and less severe neuropsychiatric behaviours and symptoms (Z = -2.33; p = 0.02); fewer depressive symptoms (Z = -2.53; p = 0.01); lower level of distress related to behavioural and psychological symptoms of dementia (BPSD) (Z = -2.49; p = 0.01) (reported by caregivers). **Cognitive functioning**: - TG group = significant MoCA gains (Z = -2.28; p = 0.02). **Quality of life:** - TG group = significant gains in self-reported quality of life (Z = -2.05; p = 0.04) Effect size (TG group): BPSD frequency and severity: d = -0.59 (full sample) and d = -0.54 (participants with mild-to-moderate NCD only). Measure of depressive symptoms: d = -0.70 (full sample) and d = -0.56 (participants with mild-to-moderate CDT only). MoCA: d = -0.37 (participants with mild-to-moderate NCD only) Self-reported quality of life: d = -0.33 (participants with mild-to-moderate NCD only). Effect size (CG group): - Medium to large effect sizes for: frequency and severity of BPSD, distress and quality of life as reported by caregivers. - MoCA: d = -0.60 (participants with mild to moderate NCD only). - Mood measurement: small effect size.	Limits: - Small sample size. One-stop recruitment. - Sample predominantly female but reflects national trend (more women in seniors’ residences). - Participation of people with severe NCD resulted in a lack of data for some of the measures (cognitive measures and perceived quality of life). Recommendations: - Future studies to better understand how gender differences may influence the benefits of non-pharmacological interventions in people with NCD. - Larger sample size to better understand how individual differences in profiles (e.g., NCD severity) may influence the benefits of horticultural activities. - Compare either different horticultural activity programs or different nature exposure activities to identify the additional benefits of more active engagement with nature via horticultural activities for people with NCD. - Inclusion of tools to detect BPSD should be considered (especially for people living with NCD and with communication difficulties). Follow-up assessments to be considered.
Bourdon and Belmin (2021)	- Walking in a garden. - 2 types of garden were present in the study: gardens enriched with stimulation modules and conventional sensory gardens.	- Walk in the garden, alone or with others. - Staff escort participants to the garden entrance. - Family members invited to use the garden with their loved ones. - For participants in the enriched gardens, a short tour of around 15 min was given at the start to introduce the enriched garden modules. The gardens were closed at night.	- Caregivers invited patients to go for a walk in the garden. - The aim was to achieve a minimum of 4 visits per week for each participant. - The walks lasted around 10 to 20 min.	- Four retirement homes in France. - Intervention over 6 months, in spring and summer.	n = 120 3 categories of participants: - Participants with no incentive to visit the garden: n = 39 - Participants encouraged to visit conventional sensory gardens: n = 41 - Participants encouraged to visit enriched gardens: n = 40 - All the residents were able to walk.	**Cognitive functioning**: Difference in total MMSE score before and after intervention (p = 0.0001): - Decrease for participants with no incentive to visit the garden: -0.25 ± 0.71. - Decrease for participants with incentive to visit conventional sensory gardens: -0.24 ± 0.73. - Improvement for participants encouraged to visit enriched gardens: +0.93 ± 0.65. **Functional and decision-making autonomy**: ADL independence: - Participants with no incentive to visit the garden: -0.05 ± 0.32 (p = 0.0001). 13% worsened, 77% no change and 10% improved (p < 0.0001). - Participants encouraged to visit conventional sensory gardens: -0.12 ± 0.24 (p = 0.0001). 37% worsened, 39% unchanged and 24% improved (p < 0.0001). - Participants encouraged to visit enriched gardens: 0.30 ± 0.35 (p = 0.0001). 100% improved (p < 0.0001).	Limits: - Pilot trial was not randomized. - Garden assignments were made according to room location (selection bias). - The frequency of visits and precise duration of use were not recorded, nor was the number of interactions with the stimulation modules in the enriched gardens. - Accompaniment by a carer or family member was not recorded, as this could have a positive influence on the results. - Caregivers didn't go to the gardens because of their heavy workload. Recommendations: none reported by the authors.
Buettner et al. (2013)	- A bike where the participant was seated at the front in a wheelchair. A staff member pedalled and steered the bike along a personalized circuit.	- The activity was carried out on an individual basis (participants accompanied by a member of staff). - Intervention took place at personalized times, i.e., at times when the participant presented the most neuropsychiatric behaviours (e.g., if the patient presented agitation every day around 3:00 pm, the activity took place around 2:45 pm).	- 2 weeks of intervention with 5 sessions per week. - Each session lasted around 30 min.	Intervention took place in 5 long-term shelters.	n = 54 - 65 years and older, MMSE score of 24 or less with neuropsychiatric behaviours of apathy, depression, anxiety or agitation. - M = 86.1 years. - 13 men and 41 women. - 40.1% NCD unspecified, 38.8% Alzheimer's-type NCD.	**Symptoms:** - Better mood among participants in the experimental group. **Behaviours:** - Reduced agitation.	Limitations: none reported by the authors. Recommendations: none reported by the authors.
Cedervall and Aberg (2010)	- Walk outside.	- For participant #1, walking was done individually or as a couple. He walked alone 3 times a week when he went to the day centre, otherwise he walked with his wife. - Participant #2 took walks alone with his dog on a daily basis.	Participant #1: walks 1–2 times a day with wife or alone for about 30 min. - Participant #2: walks on average 1 time a day, for about 1 h.	- Participant #1: walks around town to get to the day centre and the neighborhood. - Participant #2: forest walk.	n = 2 - Two men (participant #1: 74 and participant #2: 63). - Light NCD. - Lives in community housing with their spouses.	**Quality of life:** - Participant #1 reports feeling good when walking long distances. - Participant #2 reports that he feels alive and that it's good for him. **Symptom**: - Wife of participant #1 reports that her spouse looks alert and radiant when he walks. **Behaviour**: - The wife of participant #2 reports that her husband feels better when he's out walking in the woods. When he returns, he's calm and she notices that he's satisfied.	Limits: - Case study: no generalization intended. - Participants had the same marital status and similar cognitive functioning, and both had a positive opinion of physical activity. Recommendations: none named by the authors.
Cook (2020)	- Forest walks, birdhouse building, tree planting, nature photography, natural artwork, bird and tree identification, fire lighting and forest cooking.	- Activities took place in groups.	Not specified.	- Activities took place in an urban forest environment in Scotland, UK.	n = 5 - Men with a NCD and a family caregiver. - E = 52–83 years. - 4 types of NCD.	**Cognitive functioning**: - A wooded environment fostered a strong link with the changing seasons and stimulated memory and reminiscence. - Increased sense of mental well-being reported by one participant, resulting in memory retention. **Quality of life:** - Increased sense of empowerment and control had a positive impact on participants’ mental well-being and quality of life. This was closely linked to another theme that emerged from the study, namely support for activities valued by participants.	Limits: - Small number of study participants. - All participants were men. - Qualitative nature of the research. - Objectivity and the ability to generalize to larger populations may not be applicable to this study. Recommendations: - Future studies to determine whether interventions tailored to meet an individual's need to be connected to self, others and the environment affect well-being and quality of life. - We need to think about how to measure the well-being of people living with CNCT.
Edwards et al. (2013)	- Gathering in the garden with friends and family, observing the aviary, feeding the birds, watching the fish, strolling through the garden, enjoying a quiet spot with a watering hole, harvesting herbs and vegetables, watering plants.	- Outdoor activities could be carried out in a group (e.g., with other residents or members of their entourage) or alone.	Not specified.	- Outdoor activities took place in the garden built into the Magnolia house (dementia-specific unit).	n = 10 - 7 living with NCD - At the start of the study, 4 participants had severe NCD, 3 had moderate NCD and 3 had mild NCD. - 9 women and 1 man. - E = 79–90 years.	**Behaviour**: - Participants’ mean agitation score (CMAI) decreased by 46.7% (p = 0.00002) - Reduced agitation among all participants after the garden was built. **Symptoms**: - Participants’ mean depression score (SCDD) decreased by 13.3% (p = 0.01994). **Quality of life**: - Participants’ mean quality of life score (Demquol) increased by 12.8% (p = 0.00068).	Limits: - Generalization not possible (sample of only 10 people). - Causality cannot be established in the absence of a control group. Recommendations: none named by the authors.
Evans et al. (2022)	- Outdoor activities: nature walks, farm or forest activities, gardening and beach visits. - Indoor nature activities: building bird feeders, visiting animals, creating flower arrangements. - Indoor activities: museum visits, bowling, cinema - 90% of activities took place outdoors.	- Intervenants led activities that were carried out with several participants. Group activities.	- Average: 2 days a week.	- 5 sites studied: two care farms, a residential centre, a forest activity centre and a community activity program.	n = 35 - Participants: British and Caucasian. - M* = 81 years and 10 months (SD** = 7 years and 3 months). - 51% of participants were women.	**Quality of life:** - 16.9% (p < 0.001) increase in mental well-being before and after the activity. **Symptoms:** - Improved mood reported by staff.	Limitations: none named by the authors. Recommendations: none named by the authors.
Gueib et al. (2020)	- Being in the garden - Passive activities (e.g., sitting, contemplating) or active activities (e.g., walking, usual activities in the garden, etc.).	- Activities could be carried out alone or in the company of others, such as family members or members of the care team.	At least 12 cumulative hours had to be spent in the garden over a 2-week period.	- Garden located in a centre for individuals with progressive neurocognitive diseases and associated with a university hospital.	n = 34-Hospitalized patients diagnosed with Alzheimer's disease - 2 groups: the group not exposed to the garden (G-) and the group exposed to the garden (G+): G- : n = 18. Average NPI: 30.8 ± 13.6. Mean SCQ total score: 8.6 ± 3.4. Mean for SCQ Anosognosia: 0.7 ± 0.6. G+: n = 16. Mean NPI: 35.1 ± 14.4. Mean SCQ total score: 7.5 ± 2.9. SCQ body representation mean: 1.3 ± 0.8.	**Cognitive functioning:** FAB = Frontal Assessment Battery MT86-1= Montreal-Toulouse aphasia language testing protocol NPI-NH = Neuropsychiatric Inventory T0 = before using the garden and T1 = after exposure to the garden. For comparison between groups: - At T0: No significant difference between the two groups for neuropsychological assessments, MMSE, FAB, MT86-1 α, mini-GDS, NPI-NH and for SCQ scores. - At T1: Significant difference in total SCQ score between the two groups. G + (T1 SCQ = 10.41) versus G- (SCQ = 7.95), (p = 0.0079). For intra-group comparison: - At T1 compared with T0: No change in MMSE, MT86-1α and mini-GDS scores between T0 and T1 for G- and G + groups. - For both groups = significant improvement in NPI-NH total score between T0 and T1 (G + = 37 to 19 and G- = 29.5 to 15 after garden exposure). - For group G- = significant decrease in mean SCQ total score between T0 and T1 (this was associated with an increase in the anosognosia score). - For group G + = significant improvement in total SCQ score between T0 and T1. Significant improvement in the body representation aspect of the SCQ at T1 versus T0.	Limits: - Variables such as sunshine and activities in the garden have not been specifically studied, and would be worth investigating in the future. - Small sample size. - No randomization. - It is not possible to attribute all the effects observed to the use of the garden alone, since patients not exposed to the garden should have been able to go outdoors and be subjected to the same climatic conditions as the exposed group. Recommendations: none reported by the authors.
Ibsen et al. (2020)	- Outdoor activities at Norwegian farm-based day care centres (FDC). Activities = walks, plant and animal-related activities and other types of farm work.	Not specified.	- Time spent at the FDC averaged 5.8 h a day. - Time spent outdoors (summer and winter):2.2 h per day.	- Activities took place within FDC.	n = 94 - 58 men and 36 women living with NCD. - M = 75.8 years. - The majority of participants lived at home with a partner. - 67.8% had completed high school or had a college or university degree. Of these, 54.3% had mild NCD, 26.6% moderate NCD and 1.1% severe NCD.	**Quality of life**: Higher subjective quality-of-life scores among CDF participants. **Symptoms:** - Outdoor activities contributed to well-being and a better mood in people with NCD p < 0.001).	Limits: - Small sample. - Participants who did not have a caregiver visiting them at least 1x/week were excluded. - 10 researchers and assistants carried out the interviews and filled in the evaluation forms, which may have had an impact on data collection. - 6.35% of the sample did not have a diagnosis of NCD despite cognitive test scores showing mild cognitive impairment. Recommendations: none named by the authors.
Levinger et al. (2022)	- Exercise circuit for: joint amplitude, static or dynamic balance, functional movements (e.g., up and down stairs, from sitting to standing). - Adjust the level of difficulty according to the participant's abilities. - Experimental group: circuit exercises in the Senior Exercise Park - Control group: regular organized activities and leisure activities supervised by Diversional Therapist staff	- Group context (3–4 participants completing the exercise circuit). Exercises were performed individually.	12 weeks, twice-weekly intervention program. - Session length: between 1 h and 1 h 30 min A 12-week maintenance period (post-intervention) enabled participants who so wished to access the Senior Exercise Park under staff supervision. The aim was to let them be independent in what they did at the park.	- The intervention program takes place outdoors at the “Senior Exercise Park” belonging to the Leith Park residential centre in Melbourne, Australia.	n = 16 - Residents of Leith Park Aged-Care Facility. - 60 and over - Diagnosis of mild or moderate NCD (MMSE score over 10) Two randomized groups: experimental and control. - Experimental group: M = 83.3 ± 7.5 years and 87.5% were women. - Control group: M = 87.5 ± 3 years and 87.5% were women.	**Behaviours:** - After the intervention, greater social isolation among participants in the experimental group (significant difference) (p < 0.01). **Quality of life:** - No significant difference in quality of life between the two groups (p = 0.5). **Cognitive functioning**: - No significant difference between groups in MoCa score (p = 0.08). **Symptoms:** - No significant difference between groups in feelings of loneliness (p = 0.9) and depression (p = 0.04).	Limits: - COVID-19 = recruitment difficulties and reduction in the sample of participants available. - Impact of COVID-19 on session attendance, which may have limited improvement. - A longer intervention period may be needed to see significant physical and cognitive effects in people with NCD. Study not double-blinded for group assignment. Recommendations: - Assessments and interventions should be carried out by the same staff, to reduce confusion and build trust.
Liao et al. (2020)	- Use of the garden. - Various horticultural activities offered (e.g., planting flowers and herbs, watering plants, etc.).	- Varies according to residence. Some activities were carried out in groups, while others were carried out individually. - Some gardens were freely accessible by entrance and exit, while others were not.	Not specified.	- Activities took place in the gardens of 9 residences for people with CTE.	n = 42 - Participants are staff members who completed the questionnaire. - It is not indicated in the article how many people living with NCD were observed. - For observations made in connection with gardens with free access n = 7 and for gardens with restricted access n = 35.	The results presented correspond to the average obtained using this scale: 1 = really worse than usual, 2 = slightly worse than usual, 3 = same as usual, 4 = slightly better than usual and 5 = really better than usual. 1) Effects of garden visits: **Symptoms**: - Improvement in residents’ mood (mean = 3.81; SD = 0.59). - Reduced depression (mean = 3.76; SD = 0.62). **Behaviours:** - Improved social interaction (mean = 3.71; SD = 0.64) - Decreased agitation/anxiety (mean = 3.69; SD = 0.74). - Other observed behavioural aspects: Means and standard deviations indicate behaviour between the same as usual and slightly better than usual for aggression/anger, hallucinations/delusions, repetition (words/questions/activities), sleep, suspicion, wandering, abuse (physical/emotional/verbal/sexual). **Quality of life:** - Activities of daily living (mean = 3.19; SD = 0.40), indicating no change from usual. **Cognitive functioning:** - Improving residents’ spatial skills was the aspect on which the garden had the least effect (mean = 3.12; SD 0.33). - Other aspects of cognitive functioning assessed: For temporal orientation, place orientation, short-term memory, long-term memory, attention and language skills, the means and standard deviations indicate behaviour that was between the same as usual and slightly better than usual. 2) Effects of garden visits comparing groups with free access to the garden and those with restricted access: **Symptoms:** - Significant effect on mood: higher scores for the group with free access to the garden (Mann-Whitney U = 64.500, p < 0.05). **Cognitive functioning:** Significantly better effects for the open-access group in long-term memory (Mann-Whitney U = 55,000, p < 0.05), language skills (Mann-Whitney U = 49,000, p < 0.05), spatial skills (Mann-Whitney U = 35,000, p < 0.005), aggression/anger (Mann-Whitney U = 64,500, p < .05) and anxiety/agitation (Mann-Whitney U = 58,500, p < 0.05).	Limitations: none reported by the authors. Recommendations: - Determine the effect of free access to gardens on the rate of progression of cognitive impairment. - Larger samples are recommended to verify the benefits of garden visits.
MacAndrew et al. (2019)	- Walk outside.	- Individual activity. - Each participant was paired with a trained speaker and an observer.	- Walking: average 30 min. Participants walked an average of 12.3 times over a 3-week period.	- Activity took place outdoors, in the neighborhoods surrounding the CHSLD home.	n = 7 - NCD major. - 4 women and 3 men. - M = 77 years (SD =10.28) (E*** = 66–91 years). - Participants had to have a positive history of wandering with boundary transgression with a score greater than 19 on “Revised Algase Wandering Scale-Long Term Care.”	**Behaviours**: Reported by staff: - Calmer, more communicative and sociable participants with less resistance to care. - Impact on wandering behaviours: some participants continued to walk, but their intention was more meaningful and they moved to areas where group activities were taking place. Decreased walking habits for some participants, especially at night. **Symptoms**: - Participants seemed happier according to staff **Quality of life:** - Staff member: the program seems to have improved the quality of life and enriched the lives of participants.	Limits: - Small sample of 7 participants prevented statistical analysis that would have explored the effectiveness of SWP. Recommendations: none named by the authors.
Mapes (2012)	- Forest walk and outdoor picnic.	- Activities carried out in a group that includes participants with a NCD, family members, residence staff and member of a partner organization.	- A day out in the woods.	- The outings took place in 3 separate parks. - In woods and green spaces, no more than 45 min from participants’ homes. - Accessible parking, toilets and wheelchair paths in the woods.	n = 24 Hylands: n = 7, all in wheelchairs. Sheffield Park: n = 9, 4 in wheelchairs. Westonbirt: n = 8, including 5 in wheelchairs.	**Behaviours:** - Increased verbal expression. **Symptoms:** - Improved sleep. - Improved food intake. - Improved mood. **Quality of life:** - Sense of belonging. - A feeling of friendship. - Positive social encounters. **Functional and decision-making autonomy:** - Increased sense of control. **Cognitive functioning:** - Improved memory.	Limits: - Possible social desirability bias. - Relatively small sample size. - Composition of participants differs between before and after data collection (due to absences). - The results refer to qualitative analyses by the same author, but article not available. Recommendations: - Have a team or professional dedicated to taking photos/videos of walks.
Ford Murphy et al. (2010)	- Visit a garden.	Not specified.	Not specified.	- Garden in a residence with a NCD unit.	n = 34. - Male veterans of a NCD unit. - 4 participants died and 1 moved to another department. - E = 74–92 years. - M = 80.71 years. - Me**** = 80 years. - 62% of participants were able to walk unaided. Other participants used walkers or wheelchairs.	**Behaviours:** Increase in average number of garden visits = associated with an average 0.074-point decrease in agitation score (p < 0.001).	**Limits:** - Ambulant patients benefited more from their garden visits than those who were not completely ambulant, even though they had a similar number of visits. Non-ambulatory patients’ particular needs for access to the garden were not being met. - Self-selection of treatment by participants. - No control or comparison group. - Time spent in the garden was recorded in days rather than minutes. Spending 5 min in the garden was assessed as having the same impact as spending 90 min. - Visits from residents could go unnoticed if no member of staff observed the garden. Recommendations: - Include a control group and examine other possible factors in agitation levels. - Include regular visits to the garden as part of the experimental group's weekly routine. - Organize visits in a greenhouse to allow the garden to be displayed even in bad weather.
Olsson et al. (2013)	- Walking with other people, watching children play, talking to people on the street, listening to the birds sing, walking a dog, riding a bike.	- The outdoor activities carried out by the participants were varied. Some of the activities were carried out in groups, others with a loved one, and others on an individual basis.	Not specified.	- Activities took place close to participants’ homes (e.g., in their backyards or neighborhoods).	n = 11 - E = 52–81 years - Participants at an early stage of NCD (MMSE between 21–28). - Lives at home (9 lived with a relative, while 2 lived alone). - 4 people lived in a terrace house, 3 in a detached house, 4 in an apartment and 5 in a holiday cottage. - 7 participants moved around unaided, while 2 used a cane and 2 used a walker.	** Quality of life**: - Perceived sense of incapacity and reduced well-being when participants found they were no longer able to perform certain activities. - Sense of well-being associated with being able to sit outside, breathe fresh air and feel the warmth of the sun. **Cognitive functioning**: Some participants used preventive and problem-solving strategies to manage difficulties related to outdoor activities (e.g., using landmarks, stopping and thinking, using their cell phones, using different senses…).	Limits: - Small sample size. - Information collected was reported by people with NCD. Recommendations: none named by the authors.
Prolo and Sassi (2017)	- Stroll through a landscaped garden (plants, fountains, sculpture, ramps, etc.).	Not specified.	Not specified.	In a day-care centre (within a semi-public non-profit institution providing care services to a senior clientele).	n = 15 - 4 men and 11 women. - E = 69–87 years. - Diagnosis of moderate Alzheimer's disease.	**Behaviours:** - All participants (except one) felt calmer and more relaxed. **Symptoms:** - Reduced need for antipsychotic medication and sleep.	Limitations: none reported by the authors. Recommendations: none reported by the authors.
Ross et al. (2024)	- Spending time in nature and gardening.	- Activities carried out individually or in groups.	Not specified.	- Activities carried out in kind. - Outside and close to certain care residences or participants’ homes.	n = 30 - 30% of people with NCD lived with family carers, while 70% did not. - 70% were women and 30% were men. - The average age was 63.07 years. - A certain proportion of participants went to high school (6.67%), vocational school (43.33%), college (23.33%) and university (26.67%).	**Quality of life:** - A sense of well-being was associated with spending time outdoors (90.48%) and gardening (77.78%). **Behaviours:** - Spending time in nature was seen as an effective way of managing problem behaviours for some participants (n = 8). **Symptoms:** - Spending time in nature had beneficial effects on depression in some participants (n = 2). - A sense of pleasure was noted in participants who spent time outdoors (85.71%) and in those who did some gardening (88.89%). - Spending time outdoors was associated with a feeling of relaxation among participants (100%). **Functional and decision-making autonomy:** - Gardening was associated with a feeling of competence among participants performing this activity (77.78%).	Limits: - The study was conducted in Germany. - Family carers can only report on their individual experiences. These represent a subjective view of the situation. Recommendations: - The results provide only an initial, descriptive overview of caregivers’ views. - The differentiation between the type of dementia and the characteristics of the caregiving situation needs to be assessed in further research.
Smith-Carrier et al. (2021)	Gardening program (e.g., spring clean-up, planting, garden maintenance, harvesting and autumn clean-up).	- Groups of 8–10 people. - Gardening instructor accompanies them to engage them (e.g., elicit their opinions, knowledge, etc.) and explain the activities. - Participants can choose to work alone or in the company of others.	Not specified.	- Therapeutic gardening program offered at a day centre in southwestern Ontario, Canada.	n = 6 - Caucasian origin Light stage NCD.	**Symptoms**: - Decreased feelings of depression - Subjective increase in happiness and energy levels. - Reduced depressive symptoms throughout the rest of the day. **Quality of life**: - Development of strong feelings of connection and mutual support between participants. - Creation of friendships. **Cognitive functioning**: - Opportunity to continue learning.	Limitations: none reported by the authors. Recommendations: none reported by the authors.
Ura et al. (2021)	- Outdoor activities related to rice growing (e.g., rice planting, rice field maintenance, rice harvesting, etc.). - Each session included a physical activity.	Not specified.	One-hour sessions per week for 25 weeks.	On a rice paddy and a vegetable field (both about a 10-min walk from the hospital).	n = 29 Reference group: day care or residential patients: 14 people receiving usual care (M = 79.9 years) with a mean MMSE score of 18.2. 6 men and 8 women. - The Rice Care Project group comprised 15 people (M = 75.6 years). Their mean MMSE score was 20.8. 11 men and 4 women.	**Quality of life:** - Sense of connection reported by participants Significantly higher well-being scores after the rice-growing program (17.5 to 20.5). Reference group score = 17.6 to 16.5 (p = 0.017). **Symptoms**: - Positive changes reported by participants for sleep and communication more.” **Cognitive functioning**: - MMSE scores after intervention were not significantly different between the two groups (p = 0.797), no improvement in either group (20.8 to 21.6 for experimental group VS. 18.2 to 19.0 for reference group).	Limits: - Study only takes place in one region of Japan. - Small sample size. - Qualitative data from interviews were not audio recorded and were not transcribed verbatim. - Components of reference group activities (e.g., frequency, intensity and duration) could not be controlled. - Selection bias (intervention and reference groups were not homogeneous).
van der Velde-van Buuringen et al. (2021)	- Sitting outside, walking in the garden, talking, having a drink, playing a game, activities organized by the establishment (e.g., group gardening, going to the beach, going to the greenhouse), looking at the environment, singing, listening to music, feeding the animals, cutting one's nails).	- Outdoor activities in the CHSLD home garden. - Activities chosen by the patient from among those offered by the CHSLD home but carried out outside. - Participants always accompanied by someone (e.g., volunteers, family members, caregivers, recreational therapists) to facilitate the intervention. - Most activities carried out individually. - Some activities are organized for residents and carried out as a group.	- At least 30 min. - Frequency according to participants’ wishes.	- CHSLD home.	n = 20 - 20 participants living in CHSLD home with a diagnosis of moderate to severe NCD. - 16 women and 4 men - M = 85.2 years (SD = 4.92)	**Behaviours:** - Improved reminiscence reported by caregivers - Decreased agitation reported by caregivers - Development of new positive habits reported by caregivers - Significant reduction in social isolation between baseline and intervention periods, with mean scores of 5.73 (SD = 1.45) and 6.38 (SD = 1.60), respectively (p = 0.047). - Agitation: no significant change after intervention. **Symptoms:** - Improved wakefulness during the day. - Statistically significant increase in positive affect between baseline and intervention periods: mean 7.80 (SD = 2.08) and mean 8.90 (SD = 1.78) (p = 0.002). Statistically significant positive effect of negative affect between the intervention and follow-up periods, with a mean of 3.70 (SD =1.57) and a mean of 4.17 (SD =1.75) (p = 0.032). **Quality of life:** - No significant difference in social relationships after surgery.	Limitations: - Sample size was limited. - Staff shortages during the summer vacations, resulting in increased workload for the remaining caregivers. - Although caregivers stated in interviews that participants were outside more often than usual, the exact difference between the percentages of presence in the garden during the reference period and the intervention period is not known. Recommendations: none named by the authors.
White et al. (2018)	- Spend time in nature, including time spent in gardens.	Not specified.	- Every months except January were represented in the observations, but 76.6% of outings took place between May and September. - Duration: between 10 and 200 min	- Outside a retirement home. - Outdoor spaces have been extensively renovated and improved (e.g., diversified plantings: fruit trees, vegetables, as well as passive and active spaces) to encourage outdoor activities.	n = 28 - Participants with intermediate or advanced NCD.	**Symptoms**: Positive association between length of time spent outdoors and mood (p < 0, 001). - Marked improvements in mood = associated with a minimum time spent outdoors of 20 min. - Maximum mood improvement:80 to 90 min of outdoor time. - Over 80 to 90 min of exposure = no additional benefit. - Rapid reduction in benefits if exposure time exceeds 100 min.	Limitations: none named by the authors Recommendations: - Further investigation of the wide range of known green spaces that can benefit people living with NCD in the community. - Further studies needed to determine causality and investigate mechanisms, with the aim of providing specific recommendations to inform policy and recommendations on the development of facilities for people with NCD.

### Data Synthesis and Communication of Results

In order to highlight the effects of outdoor activities on MNCD, the data collected were categorized according to behaviors, symptoms, quality of life, autonomy, and cognitive functioning. The characteristics of outdoor activities relevant to the analysis were also categorized: type of activity, context, frequency, duration, and location (Table 3).

## Results

After removing duplicates, 445 publications were identified. Following analysis of titles and abstracts, 54 articles were read in full to check whether they met the selection criteria. Of these, 12 articles were selected. Following the consultation of systematic reviews and a scoping review, 10 additional articles from the reference list of these publications were added. In all, 22 publications meeting the inclusion criteria were selected (Figure 1).

Descriptive data are presented in Table 2. Studies were conducted in Australia (n = 1), Canada (n = 1), France (n = 2), Germany (n = 1), Italy (n = 1), Japan (n = 1), the Netherlands (n = 2), Norway (n = 1), Sweden (n = 1), Switzerland (n = 2), the UK (n = 4), and the USA (n = 3). Eight studies used a quantitative design, six a qualitative design, and eight a mixed design. Quantitative designs included: observational studies (n = 4), pilot trials (n = 3), and feasibility studies (n = 1). Qualitative designs included: phenomenological studies (n = 3), a descriptive observational study (n = 1), a feasibility study (n = 1), and a pilot trial (n = 1). Finally, the mixed designs included: observational studies (n = 2), pilot trials (n = 2), convergent studies (n = 2), feasibility study (n = 1), and randomized clinical trial (secondary analysis) (n = 1).

For each of the studies identified, a description of the different characteristics of outdoor activities, i.e., type, context, frequency, duration and location, as well as a presentation of their effects on participants’ quality of life, symptoms, behaviors, cognitive functioning, and autonomy, is presented in Table 3.

### Characteristics of Outdoor Activities

#### Type of Outdoor Activities

The studies identified in this scoping review present a wide variety of outdoor activities. Those identified in the greatest number were activities that took place in a garden, such as going for a walk, getting together with family, and birdwatching (Bourdon & Belmin, 2021; Edwards et al., 2013; Ford Murphy et al., 2010; Gueib et al., 2020; Prolo & Sassi, 2017; Ross et al., 2024). In some studies, the activities performed were highly diversified, as participants could choose for themselves which ones they wished to perform (Cook, 2020; Edwards et al., 2013; Gueib et al., 2020; Ibsen et al., 2020; Olsson et al., 2013). For example, some people could listen to music or just sit outside, while others preferred to feed the animals (van der Velde-van Buuringen et al., 2021). In two studies reviewed, only walking was performed as an outdoor activity (Cedervall & Aberg, 2010; MacAndrew et al., 2019). One study included both walking and picnicking (Mapes, 2012). In three studies, participants took part in plant-related activities such as gardening and horticulture (Borella et al., 2023; Liao et al., 2020; Smith-Carrier et al., 2021). One study offered participants a variety of forest-related activities, such as tree-planting, walks, preparing meals in the forest, as well as making natural works of art (Cook, 2020). One study included only a cycling activity where the person sat on a front seat and was propelled by another person (Buettner et al., 2013). Rice-growing activities were carried out by participants in one of the studies (Ura et al., 2021). In another study, the outdoor activity was physical exercise in the form of a circuit (Levinger et al., 2022). Some of the activities performed by participants were associated with farming and animals (Beerens et al., 2018; Ibsen et al., 2020). One study included both indoor and outdoor activities, with the latter ranging from flower arranging to museum visits (Evans et al., 2022). More than the majority of studies reviewed included outdoor activities where participants were active (Beerens et al., 2018; Borella et al., 2023; Bourdon & Belmin, 2021; Cedervall & Aberg, 2010; Cook, 2020; Evans et al., 2022; Ibsen et al., 2020; Levinger et al., 2022; Liao et al., 2020; MacAndrew et al., 2019; Mapes, 2012; Prolo & Sassi, 2017; Ross et al., 2024; Smith-Carrier et al., 2021; Ura et al., 2021) such as walking or gardening. In some studies, both active and inactive outdoor activities could be performed (Edwards et al., 2013; Gueib et al., 2020; Ford Murphy et al., 2020; Olsson et al., 2013; van der Velde-van Buuringen et al., 2021; White et al., 2018). Only one study included an outdoor activity where participants were inactive as they were on a seat on the front of a bicycle and were driven by another person (Buettner et al., 2013).

#### Context of Outdoor Activities

Several studies (n = 4) included group activities (Borella et al., 2023; Cook, 2020; Evans et al., 2022; Mapes, 2012). Other studies (n = 5), meanwhile, presented activities performed individually or in the presence of a caregiver (Bourdon & Belmin, 2021; Buettner et al., 2013; Cedervall & Aberg, 2010; Gueib et al., 2020; MacAndrew et al., 2019). In addition, a few studies (n = 7) included both activities performed alone and in groups (Edwards et al., 2013; Levinger et al., 2022; Liao et al., 2020; Olsson et al., 2013; Ross et al., 2024; Smith-Carrier et al., 2021; van der Velde-van Buuringen et al., 2021). Finally, six studies reported no information on this subject (Beerens et al., 2018; Ford Murphy et al., 2010; Ibsen et al., 2020; Prolo & Sassi, 2017; Ura et al., 2021; White et al., 2018).

#### Frequency and Duration of Outdoor Activities

With regard to the frequency and duration of outdoor activities, these two aspects show a fair degree of variability between studies. In terms of frequency, the activities identified in some of the studies (n = 7) took place several times a week (Borella et al., 2023; Bourdon & Belmin, 2021; Buettner et al., 2013; Cedervall & Aberg, 2010; Evans et al., 2022; Levinger et al., 2022; MacAndrew et al., 2019). In one of the included studies, participants took part in outdoor activities with variable frequency, i.e., according to personal preference (van der Velde-van Buuringen et al., 2021). One study reported the effects of a one-off outdoor activity taking place on a single day (Mapes, 2012). In terms of duration, most studies (n = 7) included activities lasting less than an hour (Borella et al., 2023; Bourdon & Belmin, 2021; Buettner et al., 2013; Cedervall & Aberg, 2010; MacAndrew et al., 2019; Ura et al., 2021; van der Velde-van Buuringen et al., 2021), while the activities reported in two studies were spread over a period of more than an hour (Ibsen et al., 2020; Levinger et al., 2022). Furthermore, outdoor activities in one of the studies ranged in duration from 10 to 200 min, with the majority (76.6%) taking place between May and September (White et al., 2018). The activities reported in one study did not specify the frequency (Gueib et al., 2020), but reported a total duration of hours for the study period. Indeed, the total activity period was reported as 12 h, spread over 2 weeks. On the other hand, five studies reported on activity programs taking place over a period of 2 weeks (Buettner et al., 2013), 3 weeks (MacAndrew et al., 2019), 6 weeks (Borella et al., 2023), 12 weeks (Levinger et al., 2022), and 25 weeks (Ura et al., 2021) respectively. Some studies did not report information about the frequency, period or duration of activities performed (Beerens et al., 2018; Cook, 2020; Edwards et al., 2013; Ford Murphy et al., 2010; Liao et al., 2020; Olsson et al., 2013; Prolo & Sassi, 2017; Ross et al., 2024; Smith-Carrier et al., 2021).

#### Location of Outdoor Activities

The majority of outdoor activities took place directly in the participants’ places of residence, whether in a nursing home (Beerens et al., 2018; Borella et al., 2023; Buettner et al., 2013; MacAndrew et al., 2019; van der Velde-van Buuringen et al., 2021), a specific unit for people living with MNCD (Edwards et al., 2013; Ford Murphy et al., 2010; Gueib et al., 2020; Liao et al., 2020), a seniors’ residence (Bourdon & Belmin, 2021; Ross et al., 2024; White et al., 2018) or their home neighborhood (Cedervall & Aberg, 2010; Olsson et al., 2013; Ross et al., 2024). Other activities took place on the premises of a day center (Prolo & Sassi, 2017; Smith-Carrier et al., 2021) or a green care farm (Ibsen et al., 2020). A few studies specified that outdoor activities took place in nature, for example in green spaces and forests (Cook, 2020; Mapes, 2012) or in a rice paddy and vegetable fields (Ura et al., 2021). Only one of the studies mentioned that the activities took place in a park (Levinger et al., 2022). For one of the studies, outdoor activities were carried out in several different locations, i.e., in a nursing home, in a forest, on a green care farm and in a community space (Evans et al., 2022).

### Effects of Outdoor Activities

#### Effects on Quality of Life and Well-Being

With regard to the effects on participants’ quality of life, several studies indicated an increase in well-being following outdoor activities (Cedervall & Aberg, 2010; Cook, 2020; Evans et al., 2022; Ibsen et al., 2020; Ross et al., 2024; Ura et al., 2021). What's more, the results of some studies specifically identified an improvement in participants’ quality of life (Borella et al., 2023; Edwards et al., 2013; MacAndrew et al., 2019). As for social relationships, other studies (n = 3) showed a positive impact of outdoor activities, notably through the creation of a sense of belonging (Mapes, 2012), positive social encounters (Mapes, 2012; Smith-Carrier et al., 2021) as well as a feeling of connection (Smith-Carrier et al., 2021; Ura et al., 2021). However, in one study, quality-of-life ratings did not increase in the experimental group compared with the control group, which performed usual indoor activities (Levinger et al., 2022). In another study, a lack of significant difference was also reported, although this was related to social relationships (van der Velde-van Buuringen et al., 2021). On the other hand, one study found a decrease in perceived well-being among participants who realized they were no longer able to carry out their usual outdoor activities (Olsson et al., 2013).

#### Symptom-Related Effects

With regard to symptoms, the majority of studies included in the scoping review (n = 12) showed favorable effects on mood (Beerens et al., 2018; Buettner et al., 2013; Cedervall & Aberg, 2010; Evans et al., 2022; Ibsen et al., 2020; Liao et al., 2020; MacAndrew et al., 2019; Mapes, 2012; Smith-Carrier et al., 2021; van der Velde-van Buuringen et al., 2021; White et al., 2018). In one of the studies reporting this effect, nuances were added in relation to the amount of time spent outdoors. Indeed, marked improvements in mood were linked to a duration of time spent outdoors of at least 20 min. The most significant benefits occurred at 80 to 90 min, and there were no additional benefits beyond this. When the duration exceeded 100 min, the benefits diminished rapidly (White et al., 2018). In the study by Liao et al. (2020), a greater improvement in mood was observed in participants with free access to the garden than in those with restricted access. A reduction in negative emotions experienced by participants, including depressive symptoms and distress, was also reported in several studies (n = 5) (Borella et al., 2023; Edwards et al., 2013; Liao et al., 2020; Ross et al., 2024; Smith-Carrier et al., 2021; van der Velde-van Buuringen et al., 2021). In addition, the results of one study indicated a better level of wakefulness in association with outdoor activities (van der Velde-van Buuringen et al., 2021). Improved sleep was also reported among participants in two studies (Mapes, 2012; Ura et al., 2021). One study showed an increase in food intake among participants (Mapes, 2012). In the study by Prolo and Sassi (2017), a reduction in antipsychotics and sleep medication was observed. In addition, perceptions of relaxation and pleasure were reported by participants who spent time outdoors (Ross et al., 2024). In relation to depression levels, one study reported no significant difference between participants in the experimental group, performing an exercise circuit in a park, and participants in the control group, taking part in usual activities (Levinger et al., 2022).

#### Behavioral Effects

With regard to behaviors, an overall positive effect on behaviors was observed in one study (needs citation). Indeed, a reduction in the quantity and severity of BPSD was found in participants in the experimental group engaged in horticultural activities (Borella et al., 2023). More specifically, in several studies (n = 7) agitation decreased in participants following the introduction of outdoor activities (Buettner et al., 2013; Cedervall & Aberg, 2010; Edwards et al., 2013; Ford Murphy et al., 2010; Liao et al., 2020; MacAndrew et al., 2019; van der Velde-van Buuringen et al., 2021). Fourteen of the 15 participants in another study reported feeling calmer and more relaxed after attending the garden over a 90-day period (Prolo & Sassi, 2017). In addition, improved verbal communication was observed among participants in three studies (MacAndrew et al., 2019; Mapes, 2012; Ura et al., 2021). Furthermore, in two studies, staff reported a decrease in resistance to care (MacAndrew et al., 2019) and reactive behaviors (Edwards et al., 2013) among participants. In another study, spending time in nature was perceived as an effective way to manage participants’ reactive behaviors (Ross et al., 2024). In addition, a decrease in wandering, especially at night, was observed, as well as an increase in walking intentions for meaningful activities (MacAndrew et al., 2019). One study reported a significant decrease in social isolation following diversified outdoor activities taking place in a garden (van der Velde-van Buuringen et al., 2021). However, in the study by Levinger et al. (2022), social isolation was higher among participants in the experimental group performing an individual exercise circuit in an outdoor park. No hypothesis was put forward by the authors to explain these results. On the other hand, there was a lack of overall effect in one of the studies, as a reduction in the quantity and severity of BPSD was observed in both participants in the experimental group and those in the control group attending or not attending the garden (Gueib et al., 2020).

#### Effects on Cognitive Functioning

Some studies highlighted the positive effects of outdoor activities on cognitive functioning during horticultural activities (Borella et al., 2023) or visits to conventional sensory or enriched gardens (Bourdon & Belmin, 2021). More specifically, in terms of memory, an improvement was reported following outdoor activities (Cook, 2020; Mapes, 2012). One study showed that outdoor activities enabled participants to use problem-solving strategies (e.g., stopping and taking a moment to think, using cues or a cell phone) (Olsson et al., 2013). In Smith-Carrier's (2021) study, participants reported that outdoor activities enabled them to learn new things. Finally, an improvement in long-term memory, language skills, and spatial skills was observed for participants who had free access to the gardens compared with those who had restricted access (Liao et al., 2020). On the other hand, several studies reported a lack of change following the realization of outdoor activities. Indeed, there was no change in cognitive abilities when participating in an exercise circuit (Levinger et al., 2022), a rice-growing program (Ura et al., 2021), or an activity involving access to a garden (Gueib et al., 2020).

#### Effects on Decision-Making and Functional Autonomy

Finally, two studies found positive effects in relation to autonomy. On the one hand, with regard to decision-making autonomy following outdoor activities, participants reported a sense of control (Mapes, 2012) and competence (Ross et al., 2024). With regard to functional autonomy, all (100%) participants who were encouraged to visit the enriched gardens, which are gardens with stimulation modules, showed an increase in functional independence compared to 24% of participants who visited conventional sensory gardens (37% worsened; 39% unchanged) and only 10% of participants who were not encouraged to visit the gardens (13% worsened; 77% unchanged) (Bourdon & Belmin, 2021). Enriched gardens were co-designed by seniors’ residence staff and an architecture team (e.g., washable canvas for paintbrushes, mirrors and colored pyramid prisms, pyramidal constructions of tactile, olfactory, and visual effects, bars for exercises, slopes and obstacles crossing, outdoor musical instruments).

#### Summary of the Results

Analysis of the results of the studies included in this scoping review shows positive effects of most outdoor activities on behaviors, symptoms, quality of life, autonomy, and cognitive functioning in older people with MNCD, but failed to identify common characteristics of outdoor activities associated with these positive effects. Regardless of the type, context, duration, frequency, or location of the outdoor activities performed, all the studies reviewed reported a positive effect on at least one of the MNDC consequences studied.

## Discussion

This scoping review identified and described the characteristics of outdoor activities and their effects on the behaviors, symptoms, quality of life, autonomy, and cognitive functioning of people with MNCD. The most frequent characteristics of the outdoor activities identified that showed positive effects were: 1) activities carried out in a garden; 2) active activities; 3) activities offering the choice of being carried out in a group or individually; 4) activities lasting less than an hour; and 5) activities taking place in the participants’ place of residence. Positive effects of outdoor activities on mood were reported in the majority of studies. Several studies also reported improved cognitive functioning, increased well-being, and reduced agitation. This scoping review also found that different types, contexts, frequencies, duration, or locations of activities are associated with beneficial effects on at least one of the consequences of MNCD, i.e., behaviors, symptoms, quality of life, autonomy, or cognitive functioning.

Firstly, both active and inactive activities show benefits in terms of the five main consequences of MNCD named above. However, more studies used active activities which, as in the general population, constitute physical activity which is largely recognized to bring positive effects on health, well-being (Cedervall et al., 2015) and mood (Junge et al., 2020). Even just walking, a generally free and accessible physical activity, leads to an improvement in mood and a reduction in BPSD (Abraha et al., 2017; Brett et al., 2016; Souto-Barreto et al., 2015).

Secondly, the results show that whether the activity was carried out in a group or individually, it brought benefits in terms of behaviors, symptoms, quality of life, autonomy, and cognitive functioning in people living with MNCD. On the one hand, group outdoor activities frequently imply social interactions that have shown positive effects on cognitive functioning, specifically memory (Haslam et al., 2010). Moreover, participation in a group social activity has been shown to improve well-being, reduce stress, and increase decision-making autonomy (Weinstein et al., 2023). Because in this scoping review individual outdoor activities were related to positive effects, it could be hypothesized that some people benefit more from individual outdoor activities and others benefit more from group outdoor activities. Thus, it is essential to support them in adapting or changing activities that meet their needs and interests and encourage their preserved abilities. In clinical and long terms care settings, supervised group activities are more frequently offered than supervised individual activities due to shortage of staff in the healthcare system (Flaherty & Bartels, 2019). In the context of aging of the population (Altman et al., 2016), when offering outdoor group activities, it appears important to remain vigilant for manifestations of discomfort in the group for some people, in which case, activities with individual supervision could be offered.

Thirdly, the location in which the outdoor activities take places does not seem to influence positive or negative effects on behaviors, symptoms, quality of life, autonomy, and cognitive functioning in people living with MNCD. However, the literature brings up that nearby streets are the most commonly used location for recreational activities among older adults (Giles-Corti & Donovan, 2002; Huston et al., 2003) due to the opportunities they offer (Liu et al., 2020). Older adults prefer to carry out activities close to where they live, because it saves them time and money, brings them closer to their friends and is more convenient (Hu, 2022). Furthermore, among older adults, proximity to friends for informal social activities is associated with a sense of satisfaction with life (Huxhold et al., 2014) and more positive affect. Thus, outdoor activities could be performed according to the preferences or convenience and still provide benefits. Finally, the greater the frequency with which older adults engage in activities with friends, the more positive feelings they experience.

The benefits on behaviors, symptoms, quality of life, autonomy, and cognitive functioning shown in this scoping review regardless of the individual/group basis, the location and the timing of outdoor activities implies that some characteristics suit some people more and other characteristics suit other people more. Moreover, there are positive effects irrespective to the decisional freedom offered (e.g., location, frequency, individual or group basis). However, because decision latitude in choosing activities improves functional status, psychological health, perceived health, and life satisfaction in people living with MNCD (Karademas et al., 2011; Vallerand et al., 1989; Weinstein & Ryan, 2011), efforts could be made to offer minimally a few options in the type, timing, location, or duration of outdoor activities. In fact, in clinical settings, for example in hospitals and long-term care settings, choices are rarely offered regarding the type of activity, when and where it is performed, and these are primarily determined by the organization to optimize institutional efficiency and convenience (Burack et al., 2012; Das, 2017). Moreover, for people living with MNCD, decision latitude in choosing activities improves functional status, psychological health, and perceived health (Karademas et al., 2011; Weinstein & Ryan, 2011). Finally, for people living in retirement homes, the ability to make choices is associated with a higher level of satisfaction with life (Vallerand et al., 1989).

This study highlights the benefits of outdoor activities on the autonomy, behaviors, symptoms, quality of life, and cognitive functioning of people living with MNCD. However, there is little information on the characteristics of activities that have the greatest effect on participants according to their personal characteristics (level of autonomy, behaviors, symptoms, quality of life, cognitive functioning). An important point is, because of cognitive deficits and often also physical limitations, outdoor activities should be adapted to the capabilities of the person with MNCD and sometimes benefit from the supervision of staff or a caregiver. In order to target the optimal intervention to be offered to people living with MNCD, which would enable a response to their needs in a personalized way, it would be interesting to better understand how the characteristics of the activities have an impact on the different spheres listed above. It would be interesting for qualitative research to explore the benefits of specific outdoor activities for a population with MNCD and with certain specific personal characteristics, while varying the following activity characteristics: type of activity, context (e.g., group vs. individual, presence of companions, structured vs. free activities), frequency, duration, location, and participants’ decision-making freedom. Such research would be useful in targeting the characteristics of activities to be integrated into programs for people living with MNCD, in order to obtain maximum benefits, according to their personal characteristics.

## Strengths and Limitations

Methodological rigor is one of the strengths of this scoping review. Firstly, the method used is based on the steps proposed by Arksey and O’Malley (2005). Secondly, the co-validation of study selection ensures the consistency of the process. The strengths of this study also include the inclusion of qualitative, quantitative, and mixed method studies. This combination enriches understanding of the effects of outdoor activities with people living with MNCD on behaviors, symptoms, quality of life, autonomy, and cognitive functioning.

A limitation of this scoping review is the identification of studies relevant to the research question. Despite consultation with the librarian, the choice of the concept of outdoor activity in the search strategy, as well as the associated keywords, may have omitted some studies. The concept of outdoor activity is very broad and includes a wide range of activities, such as outdoor sports. In the case of activities generally practiced outdoors (e.g., hiking, gardening), they are not always specified in the title, summary, or keywords that the activity takes place outdoors. It is therefore possible that certain articles, which would have been relevant to the study, were not identified. Finally, we have not discussed the stages of different health conditions accompanied by MNCD, as well as the associated symptoms across those different illnesses.

## Conclusion

This scoping review examined the benefits of outdoor activities for behaviors, autonomy, symptoms, cognitive functioning, and quality of life in people living with MNCD. Given the degenerative nature of MNCD and the current lack of curative treatments, exploring non-pharmacological interventions such as outdoor activities represents a promising avenue for maintaining and improving the condition of people living with MNCD. However, given the limited number of studies on interventions involving outdoor activities, further studies are needed to better target the specific characteristics of these activities in relation to the benefits sought. Further studies are also needed to clarify the benefits of different activity features on quality of life, behaviors, symptoms, autonomy, or cognitive functioning. Such research is essential if we are to improve the management of patients living with MNCD.

## Implications for Practice

- Collaborating with caregivers of people living with a MNCD is a key element to implement the practice of outdoor activities.
- Activities, whether active or passive, can produce one or more benefits in terms of behaviors, symptoms, quality of life, autonomy, or cognitive functioning.
- Everyone can benefit:
  - The activity bank offers options both for people whose physical condition enables them to be active and for those whose physical condition does not allow them to be.
  - The activities can also be modulated according to the stage of MNCD.
